# Management of Ectopic Thyroid in Head and Neck: Study of 8 Cases

**DOI:** 10.22038/ijorl.2021.51080.2718

**Published:** 2021-07

**Authors:** Kanhaiyalal Agrawal, P. Sai-Sradha Patro, Pradeep Pradhan, Pradipta-Kumar Parida

**Affiliations:** 1 *Department of * *Nuclear Medicine* *, All India Institute of Medical Sciences, Bhubaneswar, Odisha, India.*; 2 *Department of Otorhinolaryngology, All India Institute of Medical Sciences, Bhubaneswar, Odisha, India.*

**Keywords:** Ectopic thyroid, Thyroid scan, Management

## Abstract

**Introduction:**

To analyze the clinical features, diagnosis, management and clinical outcomes in the patients with ectopic thyroid in a tertiary care hospital.

**Materials and Methods:**

This was a retrospective study which included eight cases of ectopic thyroid presented in the outpatient clinic. Their medical records were reviewed for clinical presentation, imaging modalities used, biochemical tests and outcomes of the management was analyzed.

**Results:**

Total eight patients were included in the study, of which five were females and three were males, within the age range of 6 - 44 years. Painless neck swelling was the predominant complaint, which was observed in the seven (87.50%) patients. Total 6 (75%) patients had normal thyroid function test and 2 (25%) patients had features of hypothyroidism. Three patients underwent surgery and three patients needed hormonal replacement. Follow-up was advised for three patients. Post-treatment follow-up of all the patients was conducted for 24 months and no significant progression/recurrence/malignant transformation of the disease was seen in any of the patients.

**Conclusion:**

Being a rare disorder, timely diagnosis and management is important to avoid complications and for better clinical outcomes.

## Introduction

Ectopic thyroid is the abnormal location of the healthy thyroid tissue (1). Besides its unusual position, it can have variations in the anatomy and the vascularity compared to the normal thyroid tissue. Although the exact cause has yet not been well explored, it could be due to the abnormal descent of the embryonic thyroid tissue during the embryonic life (2). It is mostly detected over the posterior third of the tongue; however, lungs, adrenal glands, pancreas, gallbladder, ovary, and heart can be the other possible sites to be affected by the disease (3). Majority of the patients present with painless neck swelling. Noticeably, hypothyroidism is the predominant feature in the clinical practice (4). Very rarely, dysphagia, dyspnea, dysphonia are the primary complaints by the patients, depending upon the site and pressure effect over the aerodigestive tract (5). A detailed clinical history, biochemical, radiological tests, including the fine needle aspiration cytology (FNAC), are the mainstay of diagnosis for the ectopic thyroid. Contrast-enhanced CT/MRI has been found to be of much use to look for the structural integrity of the thyroid gland and to rule out the similar benign diseases which come as the differential diagnosis. Thyroglossal cyst having similar physical findings and more frequent in occurrence than the ectopic thyroid, should always be excluded from an ectopic thyroid. A thyroid scan is always considered as the confirmatory radiological test for the final diagnosis of the disease. It ensures the exact location of the ectopic thyroid tissue and its functional integrity. An indication of the surgery is very specific, and it is confined to the patients presenting with dysphagia, dyspnea, hemorrhage and malignant transformation (6). Patients need a close follow-up to rule out the malignant change in the ectopic thyroid tissue, although later is very rare. Here we have reviewed 8 cases of ectopic thyroid and discussed their clinical profile and management in a tertiary care hospital.

## Materials and Methods

This was a retrospective case series which included 8 patients of ectopic thyroid during March 2015 to June 2017 in the Department of Otorhinolaryngology and Head Neck Surgery at a tertiary care referral hospital. Patients of ectopic thyroid located at various subsidies in the head, neck were included for the study. The medical record was reviewed and clinical, radiological, pathological data was evaluated, including their respective management. For clinical data, the symptoms, signs, diagnostic procedures were evaluated along with the surgical approaches, and significant intraoperative/postoperative complications were noted in patients who underwent surgery. The data obtained was statistically analyzed (SPSSS version 20). Patients were followed in the thyroid clinic at the end of 3, 6, 12 and 24 months after the definitive management.

## Results

A total of 8 patients were included in the study, of which 5 were females and 3 were males within the age range of 6-44 years (mean 20.42 ±10.76). The demographic data and clinical profile of the patients have been demonstrated in the table1. 

**Table 1:** Clinical presentation and the cytological features in the patients with ectopic Thyroid

**Age (Year)**	**Sex (M/F)**	**Clinical** **presentation**	**Thyroid** **profile**	**FNAC**
20	M	Submental and swelling (Only thyroid tissue)	Euthyroid	Ectopic thyroid cyst
12	F	Sublingual and Suprahyoid swelling (Dual ectopic)	Euthyroid	Ectopic thyroid tissue
08	F	Submandibular swelling	Hypothyroid	Colloid cyst
23	M	Suprahyoid swelling	Euthyroid	Ectopic thyroid
44	M	Suprahyoid swelling	Hypothyroid	Ectopic Thyroid
17	F	Lingual swelling	Euthyroid	Thyroid cyst
19	F	Submental and sublingual swelling (Dual ectopic)	Euthyroid	Ectopic thyroid tissue
06	F	Suprahyoid swelling	Euthyroid	Benign ectopic thyroid


**Fig 1:**
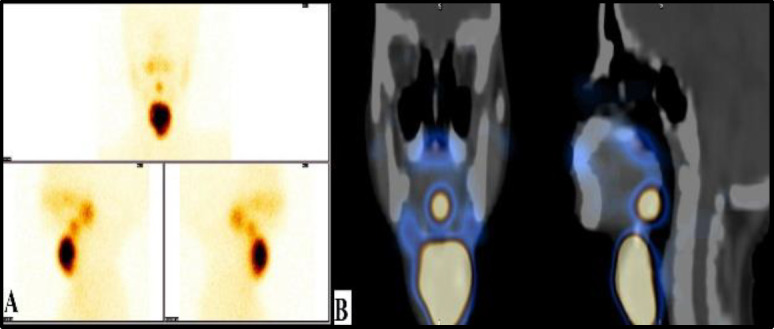
A. Tc-99m thyroid scintigraphy of a patient shows double ectopic thyroid, involving the sublingual and infrahyoid regions. B. SPECT-CT (coronal section and Sagittal section) of the neck region confirms the anatomical location of the ectopic thyroid tissue

Painless neck swelling was found to be the predominant complaint, which was observed in 7 (87.50%) patients. Only one patient presented with dysphagia and bleeding from the oral cavity. Two patients (25%) had growth retardation with respect to their chronological age besides the existing neck swelling**. **Two patients (25%) presented with multiple neck swelling, of which one had a submental-sublingual swelling and the other had submental-suprahyoid swelling. These symptoms were diagnosed as double ectopic thyroid after radioiodine scanning (Fig 1).

**Fig 2:**
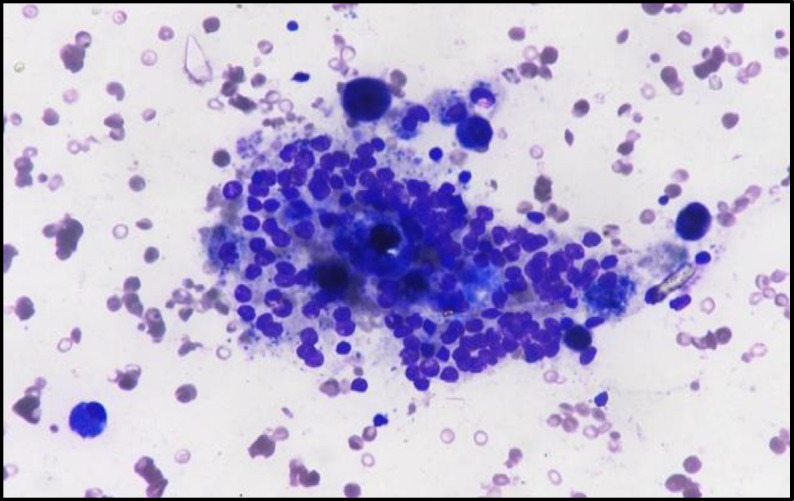
Clusters of benign follicular epithelial cells showing involutional changes along with numerous foamy and pigment laden macrophages in a background of thin colloid and RBCs (MGG, 400X) suggestive of ectopic thyroid with colloid cyst without the nuclear features of malignancy

Thyroid profile was performed in all the patients and 6 (75%) patients had normal thyroid function tests, 2 (25%) patients were hypothyroid. Ultrasonography showed that the ectopic thyroid consisting of normal thyroid tissue was identified in 4 (50%) cases, and 3 patients had cystic degeneration of the ectopic thyroid tissue (Fig 2). 

**Fig 3:**
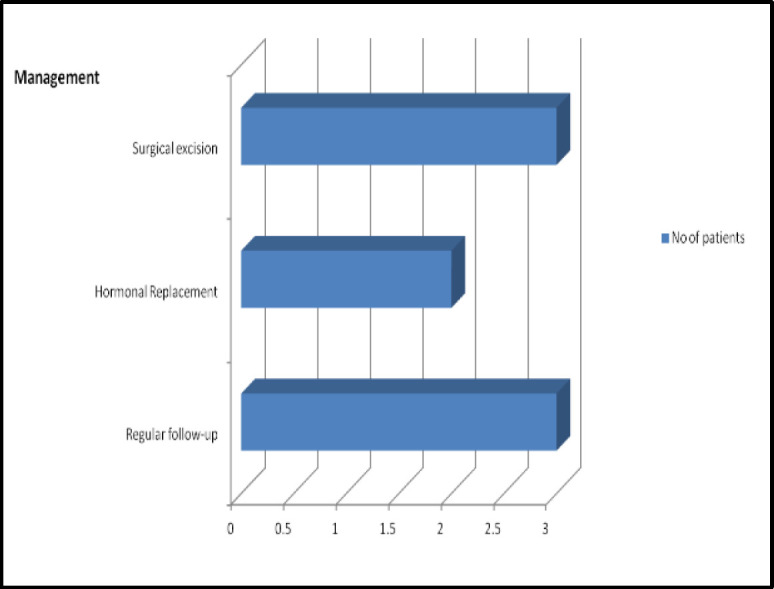
The treatment protocol of patients of ectopic thyroid

A thyroid scan was performed in each case of ectopic thyroid to find out the anatomical location and functional integrity of the thyroid tissue. The sensitivity of the thyroid scan was found to be 100% in our case series. Hormone replacement therapy was prescribed in 2 patients due to overt hypothyroidism. Of the two patients with double ectopic, one patient underwent endoscopic excision of the mass because of the dysphagia, and another was advised for the close follow-up. One patient having suprahyoid and another with sublingual ectopic thyroid underwent surgery due to the pressure symptoms. Two patients, one with dual ectopic and one with the only ectopic thyroid gland and one with a suprahyoid thyroid, were advised for close follow-up with regular work-up. The management protocol of the ectopic thyroid has been demonstrated in Fig 3. Up to 24 months of follow-up, none of the patients had any significant progression/ recurrence/malignant transformation of the disease.

## Discussion

Although the incidences of ectopic thyroid are rare in the clinical practice, still their occurrence cannot be denied at various subsidies in the head-neck region. Based on the site affected, ectopic thyroid has been classified as medial, lateral and mixed ectopic type. Medial ectopic thyroid includes the lingual, suprahyoid, infrahyoid, and endothoracis types. Lingual thyroid constitutes 90% of all the ectopic thyroid glands due to the persistent thyroglossal duct. Mixed ectopic is very rare in the clinical practice where ectopic thyroid tissue can be detected in multiple sites in the neck without involving the neck node (7). In the present case series, almost all of the detected ectopic thyroid were occupied in the medial compartment of the neck, including submental, suprahyoid and oropharyngeal location. Ectopic thyroid located in various atypical locations in the head and neck has also been described previously in the literature (8,9). Although these are usually a benign swelling found over the neck, degenerative changes can be detected over a long-standing lesion. These are similar to the normal thyroid gland with similar histopathological characteristics, which often warrant active intervention (10). In the present series, 3 patients had presented with cystic degeneration in the ectopic tissue, of which one underwent surgery and rest two were managed with conservative treatment. Neoplastic changes over the preexisting ectopic thyroid tissue are very rare (incidence 2-3%), and majorities of them are reported to be the papillary carcinoma of thyroid (11). It was also evident from the cases included in this case series that none of the ectopic thyroid tissue had any neoplastic change, which could be due to the short duration of the follow-up period. Clinical presentation of the patient mostly depends upon the site, size of the lesion, and the local pressure over the adjacent structures. Although there is great variation in the clinical symptoms in patients with ectopic thyroid, the majority of them present with a painless neck swelling (12). This has been demonstrated in the present series as well, where most of the patients 7 (87%) presented with a swelling over the anterior neck. One patient had a history of dysphagia and bleeding from the mouth at the first visit to the thyroid clinic. These symptoms such as dyspnea, heart failure, and oral bleeding as the primary complaint, depending upon the site of the ectopic thyroid tissue are very rarely reported (11,13). Similar to the normal thyroid gland, ectopic thyroid also predominantly affects the females, as observed in the present series, where 62.5% of cases were found to be females. There is no special predilection of the disease in different age groups of the patients. Patients of any age group viz. childhood, adolescence, or menopausal age can be affected where the requirement of the thyroid hormone is more. The raised thyroid-stimulating hormone (TSH) later causes enlargement of the ectopic thyroid gland (13). Different endocrine activities are monitored in the ectopic thyroid, as it could alter the thyroid function similar to the normal gland and the patient could be hypo/hyper/euthyroid. In the present series, 25% of cases had hypothyroidism and one patient had shunted growth during the initial presentation (14). Although the etiology of the ectopic thyroid has been well established in the literature, the dysgenesis in the ectopic thyroid is still unclear, in spite of the proposed gene mutations (15). As these are located in the midline in the neck, other benign and malignant lesions of the neck like thymoma, thyroglossal duct cyst, hyperplastic lymphoid tissue, lymphangioma, fibroma, lipoma, dermoid cyst, squamous cell carcinoma, minor salivary gland tumor, lymphoma, and vascular tumors should be excluded before the confirming the primary diagnosis (16,17). Although the thyroid function test, USG of the neck, and aspiration cytology are the primary investigations performed routinely for each patient of ectopic thyroid, thyroid scan/SPECT–CT has been considered as the hallmark of diagnosis providing both the anatomical location and functional integrity of the ectopic thyroid gland in the body (18,19). Keeping this in view, in the present series, both cytological and radiological (USG, thyroid scan) tests were carried out in each patient for the primary diagnosis. CT/MRI is often required in symptomatic cases to find out the anatomical detail of the lesion and to rule out the other differential diseases closely related to the ectopic thyroid (20,21). Of 8 patients, 3 patients (two patients with dual ectopic thyroid and one with suprahyoid ectopic) underwent contrast-enhanced MRI scan to evaluate, if the extension of these lesions exists and possible need for surgical excision. Management of ectopic thyroid mostly depends upon the clinicopathological profile of the patients. Asymptomatic patients with normal thyroid function are usually advised for a close follow-up in the thyroid clinic with regular USG of the neck. Medical treatment in the form of hormonal therapy has been indicated in patients with hypothyroidism, which was observed in the present series as well, where 2 patients required thyroxin supplementation due to overt hypothyroidism. Surgical treatment is only reserved for the symptomatic patients when there is the failure of the medical treatment or the swelling causing pressure symptom, hemorrhage, or suspicion of malignancy (18,19). 

In the present series, 3 patients underwent surgical excision of the mass, one patient had a history of oral bleeding and the other two had pressure symptoms during the initial presentation. Radioiodine ablation is an option among patients who are unfit for surgery and among elderly patients, although later is contraindicated in children because of its adverse effects (22). Malignant degeneration in an ectopic thyroid is observed rarely (11). In our case series, none of the patients had clinical and radiological features of malignancy till 24 months of follow-up (23,24). All the patients needed regular follow-up, irrespective of the treatment adopted, to look for the progress/degenerative changes after the definitive management (25).

## Conclusions

The incidences of ectopic thyroid are rare in the clinical practice and the predominant complaint is the midline neck swelling unless it compresses the aerodigestive tract. A complete history, clinical examination, along with the thyroid scan, is the mainstay of diagnosis. FNAC not only ensures the presence of the thyroid tissue, but it also gives information on the possible association of the degenerative changes. Management mostly depends upon the clinical-radiological profile of the patients and a close follow-up is always advised to the patients of ectopic thyroid to look for the progression/degenerative/malignant changes after the definitive treatment.
